# Disentangling Working Memory Functioning in Mood States of Bipolar Disorder: A Systematic Review

**DOI:** 10.3389/fpsyg.2017.00574

**Published:** 2017-04-26

**Authors:** Carolina Soraggi-Frez, Flávia H. Santos, Pedro B. Albuquerque, Leandro F. Malloy-Diniz

**Affiliations:** ^1^Department of Psychology, Faculty of Philosophy and Human Sciences, Federal University of Minas GeraisBelo Horizonte, Brazil; ^2^School of Psychology (CIPsi), University of MinhoBraga, Portugal; ^3^Department of Mental Health, National Science and Technology Institutes (INCT-MM), Federal University of Minas GeraisBelo Horizonte, Brazil

**Keywords:** emotion, working memory, bipolar disorder, hedonic detector, mood states

## Abstract

Working memory (WM) deficits are often reported in patients with Bipolar Disorder (BD). However, it is not clear about the nature of these WM deficits (update or serial order processes) and their association with each BD states (euthymic, mania, and depressive). This review investigated the association between BD patient's states and the functioning of WM components. For this purpose, we carried out a systematic review fulfilling a search in the databases Medline, Scopus, SciELO, and Web of Science using specific terms in the abstracts of the articles that generated 212 outcomes in the restricted period from 2005 to 2016. Twenty-three papers were selected, completely read, and analyzed using PICOS strategy. The mood episodes predicted deficits in different components of WM in BD patients (the phonological loop or visuospatial sketchpad) and were associated with different WM processes (updating and serial recall). Lower cognitive scores persist even in remission of symptoms. This result suggests that WM deficit apparently is stage-independent in BD patients. Furthermore, findings suggest that the neutral point on Hedonic Detector component of WM could be maladjusted by BD.

## Introduction

Bipolar Disorder (BD) is a severe and chronic disorder characterized by emotional and humoral dysregulation that leads to increased frequency of cognitive dysfunction and interpersonal problems (Chang et al., 2012). BD type I is characterized by one mania episode or rapid (daily) cycling episodes of mania and depression, preceded or followed by hypomanic or major depressive episodes, presenting euphoria and exaggerated behaviors such as glibness, greatness, flight of ideas, leading to social problems. In type II, at least one hypomanic episode alternates with depression phases, occurring less externalizing problems compared to mania episodes regarding to mood switching, interpersonal, and occupational problems (American Psychiatric Association, 2013). It has estimated that BD rank is the 17th cause of years lived with disability in Europe, accounting for 1.6% of the total (World Health Organization, 2016). In the United States 2.6% of adults are affected by BD (National Institute of Mental Health, 2011) and in developing countries like Brazil ~1% of the population are diagnosed with BD type I and II (Lima et al., 2005).

In addition to mood changes, patients with BD tend to show deficits in executive functions, verbal and visuospatial episodic memory, working memory (WM), verbal learning, information processing speed, sustained attention, and sensorimotor performance (Bora et al., 2009; Latalova et al., 2011; Lage et al., 2013; Loschiavo et al., 2013; Lee et al., 2014; Neves et al., 2014). Some studies have suggested more severe cognitive deficits in episodes of manic/mixed mood compared with depressive and euthymic episodes (Sweeney et al., 2000). Latalova et al. (2011) also observed that cognitive deficits tend to persist even in euthymic episodes, whereas, lower scores for verbal short-term memory and executive functions were found in both types of BD mood episodes, in tasks of WM, executive control, and verbal fluency. Some studies have shown that cognitive deficits observed in acute manic and depression episodes (Rubinsztein et al., 2000; Clark et al., 2001; Murphy, 2001), are also present in euthymic periods (van Gorp et al., 1998; Martinez-Aran et al., 2007; Wingo et al., 2009). Moreover, lower cognitive scores persist in visuospatial memory, verbal short and long-term memory, executive function, attention/processing speed, and WM, even after a long period of symptoms remission (van Gorp et al., 1998; Rubinsztein et al., 2000; Robinson et al., 2006; Martinez-Aran et al., 2007; Torres et al., 2007; McKenna et al., 2013; Ajilore et al., 2015). Besides that, first-episode BD is associated with widespread cognitive dysfunction (Lee et al., 2014), this result indicates cognitive deficits even in the earliest stages of disease and reinforces them as primary characters of BD. Symptoms of BD as anxiety and depression seem to reflect the malfunctioning of systems that provide a basis for action within a complex emotionally valenced world (Baddeley, 2013).

Among the cognitive deficits reported, WM seems to be relevant to understand the symptoms of BD. According to Miyake and Shah (1999) there are at least 10 diverse frameworks pointing different nature, structure, and functions of WM, which could diverge in terms of explanations of WM deficits. Then, it is important specifies some issues regarding this cognitive skill and its assessment that will be taken into account in the present paper. For instance, different tasks are considered measures of WM components: simple span tasks as measures of verbal and visuospatial storages, whereas, complex span tasks combine storage and attentional control (Baddeley, 1986). Currently, complex span tasks and n-back tasks are the most used WM measures (Miyake and Shah, 1999), since both require storing and maintain the information of a set of stimuli, and inhibiting interference of the signals recently presented (St Clair-Thompson and Gathercole, 2006). But there are dissimilarities, complex span tasks request keeping a serial order in mind at the same time of an ongoing cognitive task (Daneman and Merikle, 1996; Unsworth and Engle, 2007; Conway and Kovacs, 2013), while n-back tasks demand a constant updating of the relevant information (Kane et al., 2008; Wilhelm et al., 2013; Remoli and Santos, 2017). Apart from that, there are low correlations between n-back tasks and complex span tasks leading a conclusion that both evaluate different processes of WM (Kane et al., 2008; Redick and Lindsey, 2013), particularly concerning to the influence of emotional states (Ribeiro et al., submitted). A systematic review showed that different verbal WM capacities could be observed in studies that used mood induction before the completion of the task. While positive and negative stimuli seem to decrease the span task performance, no effects were found for n-back tasks (Ribeiro et al., submitted). In fact, negative mood induction can decrease WM performance during simple span and complex tasks (Spies et al., 1996; Santos et al., 2015; Soares, 2015).

In this review, WM is assumed to be a temporary information storage system that underpins the capacity for active thought by providing a cognitive workspace in which information may be temporarily maintained and manipulated (Baddeley, 2013). It is composed of multiple components, as the central executive, responsible for attentional control, and two subsidiary systems: the phonological loop and the visuospatial sketchpad, responsible for storing auditory-verbal and visual-spatial information, respectively (Baddeley et al., 2012). Subsequently, the episodic buffer was added to the initial WM model for integrating information from its multiple components with long-term memory (Baddeley, 2000). Previous studies indicated that when emotions and valenced thoughts are brought to WM they elicit somatic states (Bechara and Damasio, 2005), and the activation in brain emotional circuits, as conscious experience (LeDoux, 2000).

In fact, the connection of emotional states and working memory goes beyond the measurement instruments but in theoretical framework as well. For instance, the somatic marker hypothesis presented by Damasio et al. (1991) describes a mechanism for interaction between emotion and cognition in trial and decision-making processes (Bechara and Damasio, 2002, 2005; Bechara et al., 2005; Verdejo-García and Bechara, 2009). Damasio goes further by stating that the central executive component of working memory could be relevant in this process. As far as we know Baddeley's is the only model of WM explicitly addressing the capacity to temporary manipulate stored hedonic information. Baddeley proposed a new component, the Hedonic Detector which is understood as a neutral point that varies between positive and negative valences in response to environmental stimuli by establishing a mean value between stimulus and information retained in the WM to enable choices of future actions (Baddeley, 2007). It means that processing “hot” information (emotional content) disrupt the functioning of “cold” cognitive process, like core WM processes. Accounting this assumption, Baddeley et al. (2012) demonstrated that hedonic judgment of stimuli, such as words, pictures, and faces indeed could be influenced by the valence of an induced mood. Recently, different studies observed that mood induction procedures, e.g., listening valenced instrumental music while retrieving valenced autobiographical memories affect scores in WM tasks of health controls (e.g., Spachtholz et al., 2014; Allen et al., 2015). In this perspective, improper adjustment of the neutral point could be expressed by deficits to maintain, manipulate, and/or update information in WM. In the case of pathological affective episodes likewise in Bipolar Disorder (BD), the episodes itself could uncalibrated the hedonic detector.

Deficits in WM processing have been consistently reported in euthymic patients (MacQueen et al., 2005; Thompson et al., 2007; Daglas et al., 2015). Another study also observed that euthymic BD type I had worse performance on visuospatial tasks compared to healthy subjects (Farahmand et al., 2015). Although, there is no consensus in the literature, these findings suggest that deficits in WM could result from the intensification of emotional valences. In other words, the presence of WM deficits, could be considered a primary trace of BD, beyond state variables (Gruber et al., 2009; Kurtz and Gerraty, 2009). However, this particular issue remains controversial due to the diversity of methodologies used across the studies. In addition to the prevalence of within-subjects design, some authors rarely made the connection between theoretical models and cognitive tasks, limiting the comprehension about WM and their particular components.

In line with this argument, a review by Baddeley (2013) showed that in depressive patients negative mood influences hedonic judgment, explaining the trend of the negative perception of the situations in this clinical population. However, there is no significant evidence in respect to the influence of positive mood in hedonic detection system. Hypothetically, assuming that the neutral point in BD patients corresponds to euthymic phase, the exacerbated positive and negative valences would account for euphoric or depressed mood, respectively (Baddeley, 2007, 2013). As emotion in BD is deregulated, the study of this disorder seems to be necessary to understand the influence of improper adjustment of the hedonic detector neutral point in WM.

There are many meta-analyses which state significant neuropsychological deficits in euthymic bipolar patients (Robinson et al., 2006; Torres et al., 2007; Bora et al., 2009; Kurtz and Gerraty, 2009; Mann-Wrobel et al., 2011) and their relatives (Balanzá-Martínez et al., 2008; Arts et al., 2009; Bora et al., 2009). Most of these studies including working memory performance in bipolar patients Robinson et al. (2006), Torres et al. (2007), and Kurtz and Gerraty (2009) found that BD patients present deficits in measures of verbal working memory. In a recent review, Cullen et al. (2016) found similar results pointing that BD patients performed worse than controls in attention/working memory tasks. Bora et al. (2011) found evidences of verbal working memory deficits both in Type I and II bipolar patients. These deficits are found even in BD's relatives suggesting that these deficits are potential endophenotypes in Bipolar disorder; (Balanzá-Martínez et al., 2008; Arts et al., 2009) found evidences of worse performance in verbal working memory tasks in both BD's patients and relatives. Nonetheless these WM deficits are heterogeneous and seems to be affected by both demographic (e.g., educational level) and clinical aspects of the disorder (e.g., number of episodes) (Arts et al., 2009; Kurtz and Gerraty, 2009).

More recently, some studies using meta-analytical methodology addressed specific cognitive deficit in the bipolar patient as in theory of mind (Bora et al., 2016), social cognition (Samamé et al., 2015), and verbal fluency (Raucher-Chéné et al., 2017). Nonetheless, we did not found studies focusing in working memory deficits in bipolar disorder patients. Most of systematic review consider working memory in comparison to another cognitive functioning and some of than considered these deficits together with another executive functioning component (Robinson et al., 2006; Torres et al., 2007) and even attentional functioning (Cullen et al., 2016). Therefore, the purposes of this study are (i) to update the knowledge of working memory deficits including recent published data about working memory deficits in bipolar patients; (ii) analyze if BD patients show WM deficits, contrasting studies that used complex span tasks and n-back tasks; (iii) to investigate if different BD mood episodes predict different patterns of WM functioning, analyzing its components in each mood phase. The primary hypothesis of the present review is that working memory deficits are worsened during active phases of the disorder but remains in euthymic patients.

## Methods

This study is a descriptive and informative systematic review of the literature about the association between BD patient's states and the functioning of WM components. We used the instructions of Cochrane Foundation (Higgins and Green, 2011) to ensure the presentation of comprehensive and unbiased data. The following questions were asked to guide the review: Do patients diagnosed with BD have deficits in working memory? Are specific states of BD (mania, hypomania, depression, and euthymic) related to changes in working memory processes, such as updating and serial recall?

After the definition of the guiding questions, the following steps were taken: setting and collection of studies in databases; critical evaluation of studies, data selection and analysis, presentation, and interpretation of results (Bento, 2014).

All articles indexed in MEDLINE, Scopus, SciELO, and web of Science search databases that used, at least, one WM task in patients with BD, during a specified mood episode were included. Papers with available abstracts and written in Portuguese, English, or Spanish were considered.

Selected articles evaluated the WM in adult patients with BD, aged from 18 to 65 years old, and published until 15st July 2016. Literature reviews, case studies, and studies that referred to psychological or pharmacological interventions were excluded. Research and selection of manuscripts were done by the first author (CSF) and reviewed by the second author (FHS), with agreement between them.

For searches in databases, the terms “*transtorno bipolar*,” “*memória operatória*,” and “*emoção*”; “*trastorno bipolar*,” “*memoria operativa,” and “emoción”*; and “bipolar disorder,” “working memory,” and “emotion” were used. We found 55 papers in MEDLINE, 63 in Scopus, 1 in Scielo, and 95 in Web of Science in the first search until 15th July 2016. No studies were found in Portuguese and only one study was written in Spanish.

Among the 214 papers identified, 34 were repeated, and 20 were found in more than two databases. Based on the abstract reading, from the 160 remaining studies 25 papers were selected and 135 were excluded, due to the presented criteria: (1) 16 did not use WM tasks; (2) 42 referred to literature reviews; (3) 27 had samples whose participants were under 18- or over 65-years old; (4) eight evaluated the effectiveness of psychological or pharmacological interventions; (5) three were animal sample studies; (6) 39 had undiagnosed BD participants.

**Figure 1:**
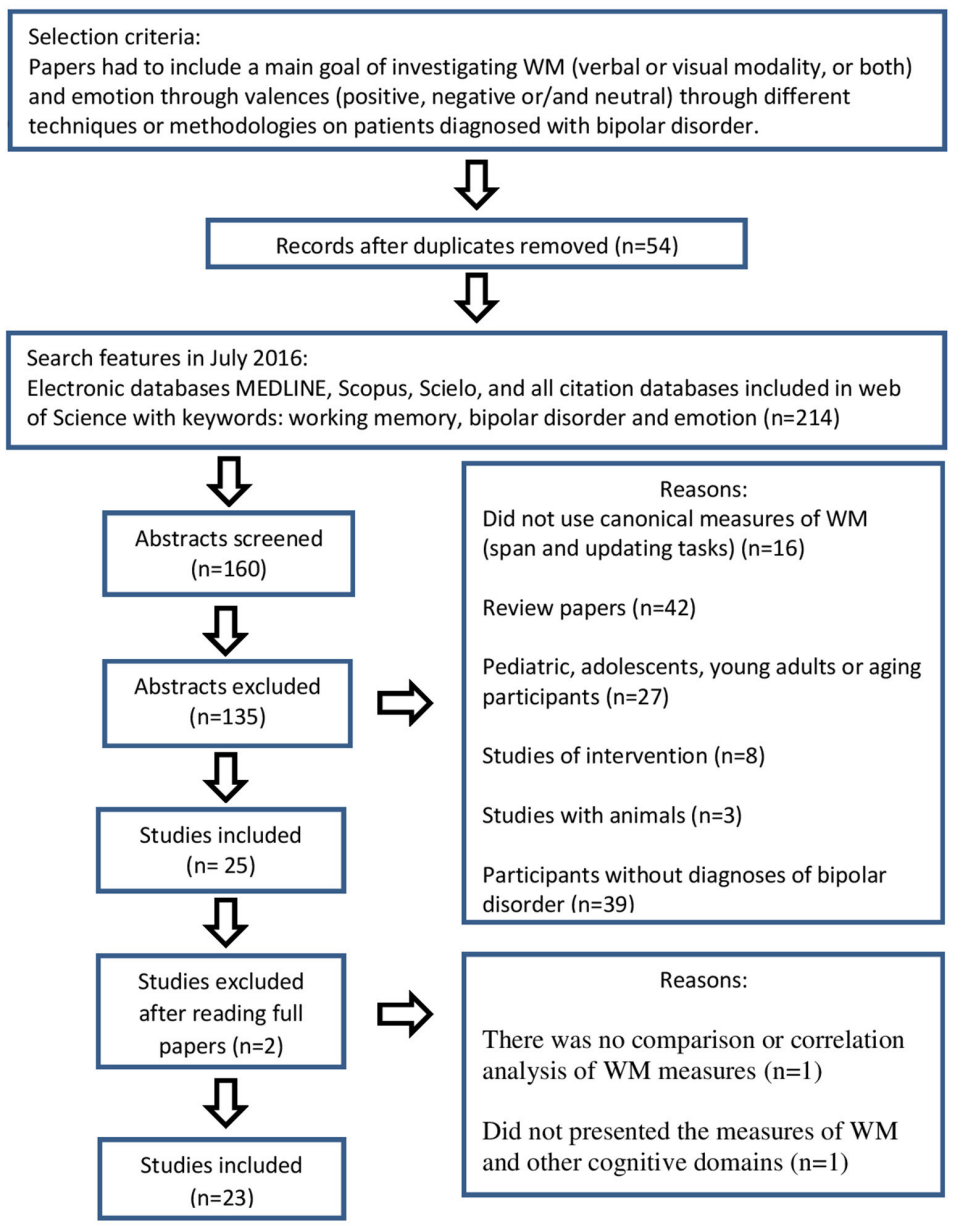
**Flowchart of search criteria and articles included in this review**. WM, working memory.

Twenty-five articles were thoroughly analyzed, being excluded two studies for the following reasons: (1) the lack of contrast between WM measures and non-social domains, which were used only as demographic control measures; (2) absence of description about the measures of WM and other cognitive domains in this session. As a result, 23 papers were discussed in this review (Figure 1).

Since most studies were methodologically unrelated to each other, carry out a meta-analysis in the present state of art would be premature, therefore we opted to systematically organize the selected articles hoping that this effort will provide new insights for researchers in order to advance in this field. Therefore, the current systematic review structured the analyses focused on the three main working memory processes (maintenance, manipulation, and updating), in respective modalities (verbal or non-verbal). The fundamental aspects of the papers were organized using the PICOS strategy, an acronym that represents the initials of the items: Population, Intervention (procedure), Comparison, Outcome, and Study Design (Bento, 2014). In this way, we used the following topics: population, type of intervention, methodological design, evaluated variables or expected results, and study design (Table 1). Based on these items we could discuss them into three sessions: “Cognitive deficits and neurobiological correlates in BD patients,” “Different patterns of cognitive processes in each mood phase,” and “Relationship among WM and other clinical features.” The first two items addressed clearly the objectives that motivated the present study and the final session account for other findings observed in the selected articles.

**Table 1:** **Summary of selected articles with have investigated WM performance in BD subjects**.

**Article**	**Population**	**Intervention**	**Comparison**	**Outcome**	**Study design**
Liu et al., 2010	*N* = 14 BD I, mean age 35.6 (10.9); *N* = 13 BD II, mean age 35.1 (9.8); *N* = 21 controls, mean age 38.3 (11.9)	Neuropsychological and behavioral assessment combined with physiological parameters	Cerebral white matter patterns and cognitive functioning correlation between groups	BD I: alterations in the right hemisphere. BD II: lower scores associated with cognitive and emotional processes. Brain alterations correlated with deficits of WM and executive function in both subtypes	Quantitative cross-sectional study
Bertocci et al., 2011	*N* = 23 unipolar depression (UD), mean age 29.74 (8.22); *N* = 18 BD I in depressive phase, mean age 31.94 (8.54); *N* = 16 controls, mean age 32.76 (6.50)	Neuropsychological and behavioral assessment combined with physiological parameters	Brain monitoring using fMRI during the tasks	Higher activity of dAMCC in UD, controls and BD I groups were found during 2-back neutral faces tasks	Quantitative cross-sectional study
Deckersbach et al., 2008	*N* = 9 BD I in moderated depressive phase, mean age 27.6 (2.8); *N* = 17 controls, mean age 25.6 (5.9)	Neuropsychological and behavioral assessment combined with physiological parameters	Sadness state induction using autobiographical memory and brain monitoring using fMRI during the tasks	BD I: greater activation in the left DLPFC and dACC in sadness condition	Prospective
Lee et al., 2013—1	*N* = 68 euthymic BD, mean age 43.9 (10.6); *N* = 38 schizophrenia in remission, mean age 44.7 (9.1); *N* = 36 controls, mean age 41.4 (9.9)	Neuropsychological and behavioral assessment	Social cognitive performance among clinical sample subgroups and controls	No significant difference in social cognitive performance between BD and control groups	Quantitative cross-sectional study
Lee et al., 2013—2	Same as Lee et al., 2013—1	Same as Lee et al., 2013—1	Social and nonsocial cognitive patterns among clinical groups	BD group had similar performance to controls in all areas. BD: more nonsocial than social deficit. Schizophrenia showed the opposite pattern	Same as Lee et al., 2013—1
Lee et al., 2013—3	*N* = 68 euthymic BD, mean age 43.9 (10.6); *N* = 38 schizophrenia in remission, mean age 44.7 (9.1)	Same as Lee et al., 2013—1	Comparison of social and nonsocial cognitive performance inter-clinical groups	Schizophrenia: more social cognitive deficits. BD: more nonsocial cognitive deficits	Idem
Levy et al., 2011	*N* = 82 euthymic BD I: 22 readmitted to the hospital, mean age 39.9 (12.7); 60 outpatient care, mean age 38.4 (11.6)	Neuropsychological and behavioral assessment	Mood state and cognitive performance before discharge and after 3 months inter-groups	Readmitted group: severe mood episodes and lower scores for executive function, attention and WM, visual and verbal episodic memory performance	Cohort prospective study
Miguélez-Pan et al., 2014	*N* = 31 euthymic BD, mean age 41.3 (11.1); *N* = 25 controls, mean age 40.40 (9.7)	Neuropsychological and behavioral assessment	Descriptive analysis of executive and functional profile and comparison of results inter-groups	BD: worse performance in flexibility, plan implementing, set-shifting and verbal fluency. No significant differences in WM	Descriptive cross-sectional study
Pomarol-Clotet et al., 2014	*N* = 38 BD I in mania state, mean age 39.74 (11.3); *N* = 38 BD in depression state - 32 BD I and 6 BD II - mean age 39.89 (10.39); 38 euthymic BD I, mean age 40 (8.7); *N* = 38 controls, mean age 39.68 (8.8)	Neuropsychological and behavioral assessment combined with physiological parameters	Brain monitoring using fMRI during cognitive the tasks in different mood phases	BD patients in depressive and manic phases showed worse performance in WM and lower activation in prefrontal dorsolateral and parietal cortex compared to controls; also in parietal cortex comparing to euthymic group. Mania group reported lower activation in left prefrontal dorsolateral cortex than euthymic group	Quantitative cross-sectional study
Roiser et al., 2009	*N* = 49 BD unmedicated in depression, majority BD II, mean age 33.6 (8.9); *N* = 55 controls, mean age 34.9 (8.1)	Neuropsychological and behavioral assessment combined with physiological parameters	Cognitive deficit inter-groups	BD: lower scores for short-term spatial memory, decision-making and insensitivity to negative feedback but not inattention, visual episodic memory and WM deficits	Quantitative cross-sectional study
Thermenos et al., 2010	*N* = 19 euthymic BD, mean age 41.1 (3.1), *N* = 18 relatives without psychiatric diagnoses—RELs—mean age 36.6 (2.6); *N* = 19 controls, mean age 39.2 (2.7)	Neuropsychological and behavioral assessment combined with physiological parameters	Brain monitoring using fMRI during the tasks	BD and RELs: alterations in frontopolar cortex and insula during WM task. Correlations between brain activity, mood and WM	Quantitative cross-sectional study
Barrett et al., 2008	*N* = 26 euthymic. BD type I, mean age 52.50 (14.17); BD type II, mean age 41.43 (9.06); *N* = 26 controls, mean age 49.75 (13.11)	Neuropsychological and behavioral assessment	Cognitive deficit inter-groups and gender; cognitive performance intra-individual correlation	BD: greater visuospatial WM and verbal fluency low score. Deficits were more detectable in men compared to women, but both had similar number of errors	Quantitative cross-sectional study
Bauer et al., 2015	*N* = 90 euthymic BD, mean age 35.18 (1.3): 59 BD I, 28 BD II, 3 BD not specified; *N* = 56 controls, mean age 36.17 (1.9)	Neuropsychological and behavioral assessment	Cognitive deficit inter-groups and intra-individual correlation	BD: no affective short-term memory and verbal fluency deficit. No significant difference in WM	Quantitative cross-sectional study
Dittman et al., 2008	*N* = 74 euthymic BD—52 BD I and 23 BD II—mean age 42.52 (12.23); *N* = 42 controls, mean age 43.02 (12.75)	Neuropsychological and behavioral assessment combined with physiological parameters	Level of homocystein inter-groups and cognitive performance and level of homocystein intra-individual correlation	BD: deficit in all cognitive tasks, including WM deficits	Quantitative cross-sectional study
Drapier et al., 2008	*N* = 20 BD I, mean age 42.7 (10.4); *N* = 20 relatives without substitute TAB for BD, mean age 43 (13.8); *N* = 20 controls, mean age 41.9 (11.6)	Neuropsychological and behavioral assessment combined with physiological parameters	Brain monitoring using fMRI during the tasks	BD: lower WM scores compared to control and relative groups. BD and relative groups: more prefrontal cortex activity	Quantitative cross-sectional study
Fleck et al., 2005	*N* = 26 BD I hospitalized in mania/mixed + psychotic symptoms, mean age 28 (7); *N* = 23 euthymic BD outpatient care, mean age 28 (7); *N* = 28 controls, mean age 28 (9)	Neuropsychological and behavioral assessment	Cognitive performance, reaction time and sensitivity perceptual correlation inter-groups	Manic BD group showed recognition effectiveness and directed-forgetting effectiveness deficit. The BD group needed more effort to encode information	Quantitative cross-sectional study
Gruber et al., 2013	*N* = 29 euthymic BD I, mean age 30.28 (8.7); *N* = 29 major depression in remission, mean age 31.32 (11.32); *N* = 30 controls, mean age 31.45 (9.3).	Neuropsychological and behavioral assessment.	Negative and positive mood maintainability capacity; and WM correlation inter-groups	BD I: affective WM deficit compared to other groups. No difference between euthymic and control groups	Quantitative cross-sectional study
Malhi et al., 2007	*N* = 10 euthymic BD I, mean age 32.4 (10.8); *N* = 10 controls, mean age 31.7 (11.9)	Neuropsychological and behavioral assessment combined with physiological parameters	Brain monitoring using fMRI during the tasks on different mood valences	Control group: increased activation in medial prefrontal cortex, medial frontal cortex and right parahippocampal gyrus during positive and negative valences induction. No difference in reaction time and accuracy	Quantitative cross-sectional study
Mullin et al., 2012	*N* = 22 euthymic BD I, mean age 31.68 (8.9); *N* = 19 controls, mean age 32.54 (6.5)	Neuropsychological and behavioral assessment combined with physiological parameters	Brain monitoring using fMRI during the tasks	BD: (i) less activation in dorsolateral prefrontal cortex, dACC, and parietal cortex without emotional distractors; (ii) increased amygdala and striatum activity before negative stimuli; and (iii) greater connection activity in dACC and amygdala after positive stimuli	Quantitative cross-sectional study
Russo et al., 2014	*N* = 64 euthymic BD—77% BD I, 14% BD II and 9.4% not specified—mean age 41.2 (10.5); *N* = 109 controls, mean age 37.9 (11.6)	Neuropsychological and behavioral assessment	Mood state, temperament and cognition inter-groups and intra-individual correlation	BD: positive correlation in level of cyclotimia and irritability with information processing, WM, reasoning and problem solving	Quantitative cross-sectional study
Thompson et al., 2007	*N* = 50 euthymic BD—44 BD I and 6 BD II—mean age 44.42 (8.6); *N* = 57 controls, mean age 44.86 (9.2).	Neuropsychological and behavioral assessment	Cognitive deficit inter-groups	BD: lower scores in Backward Digit Span. No difference in recognition task, visuospatial WM, Forward Digit Span and executive processes (verbal fluency; visual attention) between groups	Quantitative cross-sectional study
Muhtadie and Johnson, 2015	*N* = 27 euthymic BD I, mean age 35.63 (12.5); *N* = 24 controls, mean age 30.42 (11)	Neuropsychological and behavioral assessment combined with physiological parameters	Autonomic physiology and emotional reactions monitoring during the task	BD: high levels of emotional (anxiety) and autonomic (cardiovascular) reactivity, which were positively correlated	Quantitative cross-sectional study
Gvirts et al., 2015	*N* = 30 BD mildly affective symptoms, 26 BD I, 4 BD II, mean age 41.93 (12.94); *N* = 32 Borderline Personality Disorder (BPD), mean age 29.01 (9.28); matching a healthy control group	Neuropsychological and behavioral assessment	Cognitive performance inter-groups and correlation between sustained attention and cognitive measures	BD: deficits in strategy formation and planning execution time comparing to other groups; poorer utilization of strategy in the SWM task comparing to BPD. BPD: deficit in planning in comparison to all groups; in problem-solving comparing to controls. BD and BPD: deficits in sustained attention	Quantitative cross-sectional study
Sabater et al., 2016	*N* = 73 euthymic BD: 29 in lithium monotherapy (L), mean age 24.3 (9.3); 28 lithium + anticonvulsants (LA), mean age 26.8 (10.8); 16 in anticonvulsant therapy (A), mean age 28.6 (12.1); *N* = 25 controls	Neuropsychological and behavioral assessment	Comparison of cognitive performance between groups	BD-L: preserved short-term auditory memory, long-term memory, and attention. BD-A: worse performance in short-term visual memory, WM, and several executive functions. All BD patients: poorly in processing speed, resistance to interference, and emotion recognition	Quantitative cross-sectional study
McCormack et al., 2015	*N* = 99 high genetic risk (AR relatives), mean age 22.0 (4.5); *N* = 52 BD higher mood severity symptoms, 27 BD I, 25 BD II, mean age 24.6 (3.8); *N* = 78 controls, mean age 22.4 (3.9)	Neuropsychological and behavioral assessment	Comparison of cognitive performance between groups	AR: verbal reasoning and affective response inhibition deficits. BD: deficits in attention. Neither AR nor BD patients showed lower scores for general intellectual ability, WM, visuospatial or language ability. Only the BD participants showed impaired emotion recognition	Quantitative cross-sectional study

*BD, Bipolar disorder; WM, working memory; REL, relatives; dAMCC, dorsal anterior midcingulate cortex; DLPFC, left dorsolateral prefrontal cortex; dACC, dorsal anterior cingulate cortex; fMRI, functional magnetic resonance imaging*.

## Results

The *SCImago Journal* & *Country Rank* was used as an indicator of visibility in scientific domains by ranking the journals in which papers were published. The ranking for psychology, neuroscience, and medicine areas in the last 7 years were considered. Twenty-one studies were published in Q1 journals (Fleck et al., 2005; Malhi et al., 2007; Thompson et al., 2007; Barrett et al., 2008; Deckersbach et al., 2008; Drapier et al., 2008; Roiser et al., 2009; Liu et al., 2010; Thermenos et al., 2010; Bertocci et al., 2011; Levy et al., 2011; Mullin et al., 2012; Gruber et al., 2013; Lee et al., 2013; Pomarol-Clotet et al., 2014; Russo et al., 2014; Bauer et al., 2015; Gvirts et al., 2015; McCormack et al., 2015; Muhtadie and Johnson, 2015; Sabater et al., 2016), one in Q2 journal (Miguélez-Pan et al., 2014), and one in Q3 journal (Dittman et al., 2008). These quartiles also indicated that the majority of selected studies were published in higher impact factor journals.

According to the publication period of the selected articles, the first research of a direct association between emotions and WM in BD patients was performed by Fleck et al. (2005). The other studies were published in the following periods. The research studies have been mostly carried out in America, with eleven studies in the United States, eight in Europe, four of them in Asia.

Considering the first topic of analysis, most studies participants were subjects of both gender, except articles by Bertocci et al. (2011), Deckersbach et al. (2008), Malhi et al. (2007), and Thermenos et al. (2010) that only studied women. There were higher prevalence of studies with euthymic phase of BD samples, but three studies considered only depressive episodes in BD subjects (Deckersbach et al., 2008; Roiser et al., 2009; Bertocci et al., 2011). Furthermore, one paper compared a manic/psychotic symptoms group with a euthymic population (Fleck et al., 2005) and another one assessed three groups: mania, depression, and euthymic (Pomarol-Clotet et al., 2014). Articles by Liu et al. (2010) and McCormack et al. (2015) did not characterize explicitly the sample mood despite presented scores of symptoms scales and the study by Gvirts et al. (2015) comprised two subgroups with regard to affective symptoms: asymptomatic and mildly symptomatic patients. Regarding the subtypes of BD, 13 studies used BD I and II mixed groups, nine studies analyzed only BD type I subjects, and one paper compared BD type I, type II, and control groups (Liu et al., 2010).

Most studies evaluated the sample in just one mood episode in BD. Euthymic patients were predominant in the samples (Malhi et al., 2007; Barrett et al., 2008; Dittman et al., 2008; Drapier et al., 2008; Thermenos et al., 2010; Levy et al., 2011; Mullin et al., 2012; Gruber et al., 2013; Lee et al., 2013; Miguélez-Pan et al., 2014; Russo et al., 2014; Bauer et al., 2015; Muhtadie and Johnson, 2015; Sabater et al., 2016), few studies evaluated depressions phases of BD (Deckersbach et al., 2008; Roiser et al., 2009; Bertocci et al., 2011) and only one compared the performance in manic and euthymic episodes in relation to health controls (Fleck et al., 2005). All studies have used between-groups comparison, and only article by Pomarol-Clotet et al. (2014) also had a within-participants design and evaluated the same patients in three characteristics mood phases of BD.

Regarding the intervention, all studies used cognitive tests and self-reported mood scales. Seven studies (Malhi et al., 2007; Deckersbach et al., 2008; Drapier et al., 2008; Thermenos et al., 2010; Bertocci et al., 2011; Mullin et al., 2012; Pomarol-Clotet et al., 2014) also applied functional magnetic resonance imaging (fMRI) to measure brain activity. In relation to the cognitive instruments, 16 studies used span tasks (Malhi et al., 2007; Thompson et al., 2007; Barrett et al., 2008; Dittman et al., 2008; Roiser et al., 2009; Liu et al., 2010; Levy et al., 2011; Gruber et al., 2013; Lee et al., 2013; Miguélez-Pan et al., 2014; Russo et al., 2014; Bauer et al., 2015; Gvirts et al., 2015; McCormack et al., 2015; Muhtadie and Johnson, 2015; Sabater et al., 2016), and six studies chose n-back tasks (Deckersbach et al., 2008; Drapier et al., 2008; Thermenos et al., 2010; Bertocci et al., 2011; Mullin et al., 2012; Pomarol-Clotet et al., 2014), with two of them using the modified EFNBACK instrument. Only one article used a supra span task, the yes/no recognition memory test, based in verbal directed-forgetting paradigm. For the evaluation of mood symptoms, the most used instruments were: Young Mania Rating Scale (YMRS) for mania symptoms (Fleck et al., 2005; Malhi et al., 2007; Thompson et al., 2007; Barrett et al., 2008; Deckersbach et al., 2008; Dittman et al., 2008; Liu et al., 2010; Bertocci et al., 2011; Levy et al., 2011; Mullin et al., 2012; Gruber et al., 2013; Lee et al., 2013; Miguélez-Pan et al., 2014; Pomarol-Clotet et al., 2014; Bauer et al., 2015; Gvirts et al., 2015; McCormack et al., 2015; Sabater et al., 2016); Hamilton Depression Rating Scales (Fleck et al., 2005; Malhi et al., 2007; Thompson et al., 2007; Barrett et al., 2008; Deckersbach et al., 2008; Dittman et al., 2008; Liu et al., 2010; Bertocci et al., 2011; Mullin et al., 2012; Lee et al., 2013; Miguélez-Pan et al., 2014; Pomarol-Clotet et al., 2014; Russo et al., 2014; Gvirts et al., 2015; Sabater et al., 2016), Montgomery Åsberg Depression Rating Scale (Malhi et al., 2007; Roiser et al., 2009; Liu et al., 2010; Bauer et al., 2015; McCormack et al., 2015), and Beck Depression Inventory (Malhi et al., 2007; Thompson et al., 2007; Drapier et al., 2008; Muhtadie and Johnson, 2015) for depressive symptoms (Table 2).

**Table 2:** **Summary of WM tasks, cognitive tests, behavioral, and physiological measures used in the selected articles**.

**Article**	**Working memory tasks**	**Cognitive tests**	**Behavioral measures**	**Physiological measures**
Liu et al., 2010	Word retrieval test from Wechsler Memory Scale-III	Wisconsin Card Sorting Test (WCST); Test for Attention Performance (version 1.02)	Young Mania Rating Scale (YMRS), 17-item Hamilton Rating Scale for Depression (HAM-D-17), Montgomery Åsberg Depression Rating Scale (MADRS)	Fractional anisotropy (FA)
Bertocci et al., 2011	N-back (EFNBACK) task	–	HAM-D-25; YMRS	Functional magnetic resonance imaging (fMRI)
Deckersbach et al., 2008	N-back task	–	HAM-D; YMRS	fMRI
Lee et al., 2013	MATRICS Consensus Cognitive Battery (MCCB)	Facial affect recognition task; Mayer-Salovey-Caruso Emotional Intelligence Test (MSCEIT); Empathic accuracy task; The Awareness of Social Inference Test, Part III (TASIT); Self-referential memory task	HAM-D; YMRS	–
Levy et al., 2011	Digit Span subtest from the Wechsler Adult Intelligence Scale—Third Edition	Trail Making Test (TMT); Controlled Oral Word Association Test (COWAT); Stroop Color-Word Interference Test; WCST; Wechsler Abbreviated Scale of Intelligence (WASI); Letter and Symbol Cancellation Task; California Verbal Learning Test II—Short Form; Logical Memory from Wechsler Memory Scale-R; Rey Complex Figure test (RCFT)	Beck Depression Inventory (BDI-II); YMRS.	–
Miguélez-Pan et al., 2014	WAIS-III Digits Forward; WAIS-III Digits Backward subtest	Token Test (TT); WAIS-III Vocabulary subtest; WCST-64; WAIS-III Similarities subtest; TMT; Tower of London-Drexel University; FAS; Five Point Test (5PT); Stroop Color and Word Test; Frontal Assessment Battery (FAB)	Global Assessment of Functioning (GAF); HAM-D; YMRS	–
Pomarol-Clotet et al., 2014	N-back task	–	YMRS; HAM-D	fMRI
Roiser et al., 2009	Cambridge Neuropsychological Test Automated Battery (CANTAB): Spatial Span, Spatial Working Memory test (SWM)	CANTAB: Intra-dimensional/Extra-dimensional Set-Shifting (ID/ED), Spatial Recognition Memory, Pattern Recognition Memory; Delayed Match to Sample, Rapid Visual Information Processing (RVIP), Cambridge Gamble task, Affective Go/No-go test (AGN), Probabilistic Reversal Learning	MADRS; Inventory of Depressive Symptomatology	–
Thermenos et al., 2010	Two-Back Working Memory Task	Control CPT-X Task; Vocabulary and Block Design subtests of the WAIS-R; reading subtest of the WRAT-R	Profile of Mood States (POMS)	fMRI
Barrett et al., 2008	CANTAB: SWM	CANTAB: Stocking of Cambridge test (SoC); (ID/ED); Set–Shifting task	HAM-D; YMRS	–
Bauer et al., 2015	Cognition in Affective Disorders (BAC-A): Digit Sequencing Task	BAC-A: Token Motor Task, Symbol Coding, List Learning, Category In-stances, Controlled Oral Word Association Test (F and S-words), Tower of London, Emotion Inhibition Test, affective auditory verbal learning test	GAF; MADRS; YMRS	–
Dittman et al., 2008	Wechsler Adult Intelligence Scale III (WAIS-III): Letter-Number Sequencing Subtest (LNST)	TMT; Repeatable Battery for the Assessment of Neuropsychological Status Form A (RBANS)	HAM-D; YMRS	–
Drapier et al., 2008	N-back working memory task	Baseline attention task	BDI; Altman Self-Rated Mania Scale (ASRM)	fMRI
Fleck et al., 2005	Yes/no recognition memory tests	CPT	YMRS; HAM-D; Scale for the Assessment of Positive Symptoms (SAPS)	–
Gruber et al., 2013	Wechsler Adult Intelligence Scale-Fourth Edition (WAIS-IV): LNST; Affective Working Memory Task	Shipley Institute of Living Scale (SILS)	YMRS; Inventory of Depressive Symptomatology-Clinician Rating (IDS-C)	–
Malhi et al., 2007	Delayed-response working memory paradigm based on the Sternberg memory task	Extracted visual stimuli of Lang Affective Norms for English Words (ANEW) database	HAM-D-17; YMRS; MADRS; GAF; BDI	fMRI task with implicit affective content
Mullin et al., 2012	EFNBACK	–	HAM-D-25; YMRS	fMRI
Russo et al., 2014	MCCB	–	TEMPS-A; HAM-D; Clinician Administered Rating Scale for Mania (CARS-M)	–
Thompson et al., 2007	Digits Forwards; Backwards Digit Span	Self-Ordered Pointing Task –modified version (SOPT); CANTAB; Executive functions and WM tasks: Stroop, initial letter; FAS, TMT	HAM-D; YMRS; BDI ASRM	–
Muhtadie and Johnson, 2015	Automated Symmetry Span Task	–	Self-Reported Emotions; BDI–SF; ARSM	Cardiovascular Physiology
Gvirts et al., 2015	CANTAB: SWM	CANTAB: RVIP, CANTAB's version of the Tower of London task (ToL), ID/ED	HAM-D-17, YMRS, GAF; clinical global impression (CGI)	–
Sabater et al., 2016	Wechsler Memory Scale-Revised (WMSR)	WAIS-III, digit span; TMT-A and B; RCFT; WCST; Tower of Hanoi (TOH-4); Stroop color word test; FAB; Copy RCFT; Eye Test	Visual Analog Scale and the Spanish Version of the Chinese Polarity Inventory; HAM-D; YMRS; CGI-BP	–
McCormack et al., 2015	RBANS; WAIS-III: digit span task and letter–number sequencing tasks	Wechsler Abbreviated Scale of Intelligence; RBANS subscales; CANTAB: ID/ED, SoC, AGN; Ekman 60-Faces emotion recognition test; Awareness of Social Inference Test -A	Family Interview for Genetic Studies (FIGS); Kiddie Schedule for Affective Disorders and Schizophrenia for School-Aged Children – Bipolar Disorder version (K-SADS-BP); K-SADS (WASH-U-KSADS); Diagnostic Interview for Genetic Studies (DIGS); MADRS; Bipolar Depression Rating Scale (BDRS); YMRS; Children's Depression Inventory (CDI)	–

*References of the instruments cited in the Table are available on the original articles*.

Methodologies of selected studies were characterized by the comparison of cognitive variables and mood episodes between clinical and control groups. Seven articles carried just a comparison of cognitive profile between groups (Fleck et al., 2005; Thermenos et al., 2010; Levy et al., 2011; Miguélez-Pan et al., 2014; Pomarol-Clotet et al., 2014; McCormack et al., 2015; Sabater et al., 2016). In addition, eight studies evaluated correlation between variables, such as cognitive pattern and brain activation pattern (Liu et al., 2010), gender and cognitive pattern (Barrett et al., 2008), functionality, and cognition (Bauer et al., 2015), homocysteine levels and cognitive performance (Dittman et al., 2008), reaction time and perceptual sensitivity (Fleck et al., 2005), humor and WM (Gruber et al., 2013; Russo et al., 2014), and cognitive pattern (Gvirts et al., 2015). Two of the seven fMRI studies stand out, one described brain activation in different mood phases of BD (Pomarol-Clotet et al., 2014) and the other assessed brain effect of mood induction (Malhi et al., 2007). The article by Muhtadie and Johnson (2015) differ from others because combined evaluation of autonomic variables during WM performance (Table 1).

The results showed different brain patterns activity during the performance of WM tests. Most of the neuroimaging studies used n-back tasks to evaluate WM processing. Studies that chose the n-back paradigm found low left dorsal anterior midcingulate cortex (dAMCC) activation in depressive phase of BD type I group (Bertocci et al., 2011), low dorsolateral prefrontal and parietal cortex activation in depression and manic phases (Pomarol-Clotet et al., 2014), and low activity of dorsolateral prefrontal cortex, dorsal anterior cingulate cortex (dACC) and parietal cortex in euthymic group (Mullin et al., 2012). However, some studies have shown conflicting results, as greater prefrontal cortex activation in BD patients and their relatives (Drapier et al., 2008) as well as increased dorsolateral prefrontal cortex and dAMCC activity in episodes of depressed mood (Deckersbach et al., 2008). Low activations in prefrontal cortex in span tasks studies were also confirmed (Malhi et al., 2007; Liu et al., 2010; Thermenos et al., 2010). Article by Liu et al. (2010) indicated that BD type I subjects tend to show lateralized alterations in the right brain hemisphere, while BD type II presented more distributed deficits. In addition, BD patients and their relatives showed frontopolar cortex and insula brain alterations (Thermenos et al., 2010) and euthymic BD subjects exposed to positive and negative valences reported less activation in medial prefrontal cortex, medial frontal cortex, and right parahippocampal gyros (Malhi et al., 2007).

Regarding the cognitive performance of participants in WM tasks, 11 studies demonstrated lower performance in BD patients compared to control groups. Three studies involving processing of visuospatial information (Fleck et al., 2005; Barrett et al., 2008; Gvirts et al., 2015); six papers concerning auditory verbal processing (Dittman et al., 2008; Levy et al., 2011; Gruber et al., 2013; Lee et al., 2013; Pomarol-Clotet et al., 2014; Sabater et al., 2016); and one article studying updating of visuospatial information (Drapier et al., 2008). Concerning effect sizes reported on such articles we decided to analyze mean effect size on studies that produced lower scores for working memory performance. This descriptive analysis showed an average effect size of 0.39 to studies that involved processing of visuospatial information; and 0.41 to the papers concerning auditory verbal processing. The only paper that used measures of updating visuospatial information presented a effect size of 0.33. We can conclude that all the average effect sizes are low (Cohen, 1988). There are also nine papers (Malhi et al., 2007; Deckersbach et al., 2008; Drapier et al., 2008; Liu et al., 2010; Thermenos et al., 2010; Bertocci et al., 2011; Mullin et al., 2012; Pomarol-Clotet et al., 2014; Muhtadie and Johnson, 2015) that reported a variety of neurobiological changes in BD patients (e.g., prefrontal cortex activation or dorsal anterior cingulate cortex; see Table 3).

**Table 3:** **Summary of WM performance and neurobiological changes in BD subjects in selected articles categorized according WM processes and modalities**.

**Article**	**Performance in WM tasks**	**WM processes and modalities**	**Effect size**	**Neurobiological alterations**
Barrett et al., 2008	↓	Processing of visuospatial information	0.58 (r)	–
Fleck et al., 2005	↓	Processing of visuospatial information	0.23 (η)	–
Gvirts et al., 2015	↓	Processing of visuospatial information	0.21 (d)	–
Muhtadie and Johnson, 2015	−	Processing of visuospatial information	0.76 (d)	Autonomic changes associated with emotional reactivity during cognitive tasks
Roiser et al., 2009	=	Processing of visuospatial information	−0.16 (d)	–
Russo et al., 2014	WM performance was associated with cyclothymic levels and irritability in euthymic episodes	Processing of visuospatial and auditory-verbal information	0.54 (r)	–
Bauer et al., 2015	=	Processing of auditory-verbal information	–	–
Dittman et al., 2008	↓	Processing of auditory-verbal information	0.41 (d)	–
Gruber et al., 2013	↓	Processing of auditory-verbal information	0.26 (η)	–
Lee et al., 2013	↓	Processing of auditory-verbal information	0.61 (η)	–
Lee et al., 2013	↓	Processing of auditory-verbal information	0.61 (η)	–
Levy et al., 2011	↓	Processing of auditory-verbal information	0.56 (d)	–
Liu et al., 2010	−	Processing of auditory-verbal information	−	BD I: lateralized changes in the right hemisphere and more cognitivedeficits. BD II: more distributed deficits associated with cognitive and emotional processes
McCormack et al., 2015	=	Processing of auditory-verbal information	−	–
Miguélez-Pan et al., 2014	=	Processing of auditory-verbal information	−0.29 (d)	–
Sabater et al., 2016	↓ BD patients in anticonvulsant therapy	Processing of auditory-verbal information	0.16 (η)	–
Thompson et al., 2007	↓	↓ auditory information updating/ ↓ processing of auditory-verbal information	0.39 (d)	–
Bertocci et al., 2011	−	Updating visuospatial information	0.75 (d)	↓ dAMCC activation with neutral stimuli in depressive episodes
Deckersbach et al., 2008	−	Updating visuospatial information	0.07 (d)	↑ DLPFC and dACC activation when negative valence stimuli were presented
			0.03 (r)	
Drapier et al., 2008	↓	Updating visuospatial information	0.33 (η)	↓ prefrontal cortex activation
Malhi et al., 2007	−	Updating visuospatial information	−	↓ prefrontal cortex activation
Mullin et al., 2012	−	Updating visuospatial information	−	↓ prefrontal cortex activation
Pomarol-Clotet et al., 2014	↓ manic and depressive episodes	Updating visuospatial information	−	Parietal alterations in manic and depressive episodes
Thermenos et al., 2010	−	Updating visuospatial information	1.05 and 1.33 (d)	Frontopolar cortex and insula alterations brain

*WM, working memory; dACC, dorsal anterior cingulate cortex; dAMCC, left dorsal anterior midcingulate cortex; DLPFC, left dorsolateral prefrontal cortex; BD, bipolar disorder; d, Cohen's d; n, Eta-squared; r, Pearson's correlation; =, no significant differences*.

However, four studies found no difference between groups (Roiser et al., 2009; Miguélez-Pan et al., 2014; Bauer et al., 2015; McCormack et al., 2015). Article by Pomarol-Clotet et al. (2014) distinguished cognitive profiles according to the mood phases of patients, showing that manic or depressive episodes in BD reported worse performance in WM tasks compared with euthymic and controls groups. However, WM deficits (Thompson et al., 2007; Drapier et al., 2008) and other cognitive low scores can also be observed in euthymic phase, for example, visuospatial WM (Barrett et al., 2008), short-term non-affective memory (Bauer et al., 2015), unsocial cognition (Lee et al., 2013), flexibility, plan implementing, set-shifting (Miguélez-Pan et al., 2014), processing speed, resistance to interference and emotion recognition (Sabater et al., 2016), and verbal fluency (Barrett et al., 2008; Miguélez-Pan et al., 2014; Bauer et al., 2015). In addition, WM performance was associated with cyclothymic levels and irritability in euthymic episodes (Russo et al., 2014; Table 3).

Euthymic patients during conducted span tasks showed deficits in several WM components, such as auditory information updating (Thompson et al., 2007), simultaneous processing of visuospatial information (Barrett et al., 2008), and processing of auditory-verbal information (Thompson et al., 2007; Dittman et al., 2008; Levy et al., 2011; Gruber et al., 2013; Lee et al., 2013). BD type I patients also reported lower scores for updating visuospatial information during the n-back task (Drapier et al., 2008).

Some researches of euthymic samples used auditory-verbal complex span tasks (Miguélez-Pan et al., 2014; Bauer et al., 2015; Sabater et al., 2016). Nevertheless, clinical groups performed similarly to controls in processing auditory information (Miguélez-Pan et al., 2014; Bauer et al., 2015). Article by Roiser et al. (2009) also did not found deficits to perform a visuospatial span task in depressive episodes in BD patients during washout of psychotropic medications.

A prevalence of quantitative cross-sectional methodology experiment designs were characterized in selected articles (Fleck et al., 2005; Malhi et al., 2007; Thompson et al., 2007; Barrett et al., 2008; Dittman et al., 2008; Drapier et al., 2008; Roiser et al., 2009; Liu et al., 2010; Thermenos et al., 2010; Bertocci et al., 2011; Mullin et al., 2012; Gruber et al., 2013; Lee et al., 2013; Pomarol-Clotet et al., 2014; Russo et al., 2014; Bauer et al., 2015; Gvirts et al., 2015; McCormack et al., 2015; Muhtadie and Johnson, 2015; Sabater et al., 2016). Only three studies used other experimental designs, such as cohort study (Levy et al., 2011), descriptive study (Miguélez-Pan et al., 2014), and prospective study (Deckersbach et al., 2008) (Table 1).

## Discussion

The aims of the present systematic review were (i) update the knowledge about working memory deficits in BD; (ii) to analyze if BD patients show WM low scores, contrasting studies that used complex span tasks and n-back tasks, and (iii) to investigate if different BD mood episodes could predict different patterns of cognitive processes based on the WM components of the tasks in each mood phase. Considering the first objective our study found similar results to previous systematic review and meta-analyses pointing to working memory deficits in Bipolar patients (Robinson et al., 2006; Torres et al., 2007; Kurtz and Gerraty, 2009; Bora et al., 2011; Mann-Wrobel et al., 2011). Most of these reviews pointed to verbal working memory deficits in BD (Robinson et al., 2006; Torres et al., 2007; Kurtz and Gerraty, 2009). These findings could be explained by the fact that those reviews considered only digit span measures. In our review, we included both verbal and visuospatial working memory measures. In agreement with previous reviews, we found that both verbal and visuospatial working memory are impaired in BD (Bora et al., 2011; Mann-Wrobel et al., 2011).

Considering objective (ii) and (iii) they were respectively presented in Sections Introduction and Methods. This review seems to be necessary because individual results of studies are confusing, due to the fact that there are three emotional phases in BD (euthymic, euphoric, and depressed) and different WM mental operations (serial recall and update), which are expressed in at least two different modalities (phonological and visuospatial) (Table 4). For this purpose, after the selection of the articles according to the inclusion and exclusion criteria, a systematic review was carried out. Twenty-three studies in the restricted period 2005–2016 were found using different approaches as cognitive, behavioral, neuroimaging, and measures of autonomic responses. The study also convey the possibility of Bipolar Disorder as model for the study of the new component of WM, the Hedonic Detector, for this reason the relationship among WM and other clinical features in the final section.

**Table 4:** **Summary of mental WM operations and phonological and visuospatial modalities in three emotional phases in BD presented in the selected articles**.

**Modality/task**	**Euthymic**	**Depressive**	**Manic**
Verbal n-back	–	–	–
Visuospatial n-back	Malhi et al., 2007; Drapier et al., 2008; Mullin et al., 2012	Deckersbach et al., 2008; Pomarol-Clotet et al., 2014	Pomarol-Clotet et al., 2014
Verbal span	Thompson et al., 2007; Dittman et al., 2008; Levy et al., 2011; Gruber et al., 2013; Lee et al., 2013; Sabater et al., 2016^a^	–	–
Visuospatial span	Fleck et al., 2005^b^; Barrett et al., 2008; Gvirts et al., 2015^c^	–	–

a *BD anticonvulsant therapy*.

b *Supra span task*.

c *Mildly affective symptoms*.

### Cognitive deficits and neurobiological correlates in BD patients

Regarding the first aim of this review, statistically significant differences in WM performance between groups were found in most studies (Fleck et al., 2005; Thompson et al., 2007; Barrett et al., 2008; Deckersbach et al., 2008; Dittman et al., 2008; Drapier et al., 2008; Levy et al., 2011; Gruber et al., 2013; Lee et al., 2013; Pomarol-Clotet et al., 2014; Gvirts et al., 2015; Sabater et al., 2016). These results confirm that BD patients tend to have lower WM performance (MacQueen et al., 2005; Thompson et al., 2007; Lee et al., 2014; Daglas et al., 2015). According to our review both modalities are disrupted, deficits in auditory-verbal information processing were reported in six selected articles (Thompson et al., 2007; Dittman et al., 2008; Levy et al., 2011; Gruber et al., 2013; Lee et al., 2013; Sabater et al., 2016) and visuospatial low scores in five other studies (Fleck et al., 2005; Barrett et al., 2008; Drapier et al., 2008; Pomarol-Clotet et al., 2014; Gvirts et al., 2015). Our analysis also showed that the effect sizes of the studies that reported deficit on BD patients on several measures of working memory were small, either on updating or maintenance measures. These effect sizes were 0.33, 0.39, and 0.41, respectively, for updating visuospatial information, processing of visuospatial information, and processing of auditory verbal information. Together with results reported on the papers that showed neurobiological changes of BD patients during the experiments, these results clearly conclude that BD affects both working memory performance and brain functioning.

According to a recent meta-analysis, there is no consensus in the literature defining if cognitive deficits are somatic markers or a consequence of mood episodes in BD (Daglas et al., 2015). Previous researches have shown that information processing, visual episodic memory, and verbal WM deficit were associated with clinical expressions of BD, since it was not observed in first-degree relatives. Therefore, these cognitive lower performances in BD were not associated with genetic susceptibility (Bora et al., 2009). A review by Boland and Alloy (2013) showed that sleep disruption present in BD patients can also be either a predisposing factor or worsening neurocognitive deficits throughout the illness course, resulting in sustained functional deficit, despite the remission of mood symptoms.

Other studies also claimed that the level of neuropsychological deficits in BD was influenced by anti-psychotics and mood stabilizers (Donaldson et al., 2003; Savitz et al., 2008), weakening the hypothesis of WM as a somatic marker of BD. Most studies in the present review used medicated samples, except the article by Roiser et al. (2009) that apparently did this variable. Although, the participants were taking psychotropic medication during assessments, most of the studies of euthymic samples reported significant WM deficits (Thompson et al., 2007; Barrett et al., 2008; Dittman et al., 2008; Drapier et al., 2008; Levy et al., 2011; Gruber et al., 2013; Lee et al., 2013). Then, at least in these cases, results do not satisfy the state-independence criterion mentioned by some authors as a key factor in assessing potential endophenotypes of BD (Hasler et al., 2006). Also, the results suggested that WM can be a susceptibility biomarker in BD patients rather than a state-dependent variable of the disorder (Gruber et al., 2009; Kurtz and Gerraty, 2009).

Despite neurobiological correlates of WM are not clearly elucidated in literature, there is evidence of the association between neuropsychological and morphological factors in BD, which support WM deficits as a possible endophenotype of BD (Glahn et al., 2010). Based on 12 studies, a meta-analysis conducted by Lee et al. (2014) showed generalized neuropsychological deficits since the first episode of the disorder. In theory, patients with BD due to the constant changes in both emotional valences; would have an uncalibrated Hedonic Detector. Consequently it would cause deficits in WM as predicted in Baddeley's model (Baddeley, 2007). Besides, in the current review, some studies observed social cognitive deficits affecting emotional response, which is an indirect evidence of dysfunctional Hedonic Detector.

Although, assessing WM deficits in people at high genetic risk of developing BD had not been an objective of this study, it was observed that some authors considered heritability (Drapier et al., 2008; Thermenos et al., 2010; McCormack et al., 2015). Relatives without psychiatric diagnoses did not report significant deficits in WM, but they showed lower scores for verbal reasoning and affective response inhibition (McCormack et al., 2015). In contrast, relatives as BD patients also showed alterations in prefrontal cortex activity and insula during WM task (Drapier et al., 2008; Thermenos et al., 2010). These results show that WM deficits are not only state variables, but seem to be primary character of the disease (Gruber et al., 2009; Kurtz and Gerraty, 2009).

However, based on the reviewed articles there is insufficient data to conclude that WM deficits are present since the onset of disease. Only one study considered population between 18 and 25 years old and it did not show lower WM scores in BD patients (McCormack et al., 2015). Among the eight researches that studied sample aged ranging from 26 to 35 years, three articles showed deficits in WM (Fleck et al., 2005; Gruber et al., 2013; Sabater et al., 2016) and four described neurocognitive deficits in BD patients (Malhi et al., 2007; Deckersbach et al., 2008; Bertocci et al., 2011; Mullin et al., 2012). Ten out of fourteen studies found poor WM performance or other neuropsychological deficits in middle age sample (Thompson et al., 2007; Barrett et al., 2008; Dittman et al., 2008; Drapier et al., 2008; Liu et al., 2010; Thermenos et al., 2010; Levy et al., 2011; Lee et al., 2013; Pomarol-Clotet et al., 2014; Gvirts et al., 2015). There was no study with sample above fifty six years old.

### Different patterns of cognitive processes in each mood phase

The second objective of this review was to investigate if diverse BD mood would predict a different WM pattern. Updating deficits of visuospatial information were found in depressive BD patients assessed by n-back tasks (Deckersbach et al., 2008; Pomarol-Clotet et al., 2014) and in mania phases (Pomarol-Clotet et al., 2014). Despite WM alterations have been reported during mood episodes of BD, it is suggested that deficits in WM persist during remission of symptoms (MacQueen et al., 2005; Thompson et al., 2007; Daglas et al., 2015; Farahmand et al., 2015).

According to neurophysiological studies in euthymic phases of BD, patients presented lower prefrontal cortex activation during visuospatial n-back tasks (Malhi et al., 2007; Drapier et al., 2008; Mullin et al., 2012). Mullin et al. (2012), for example, found lower dorsal anterior cingulate cortex (dACC) activity during EFNBACK task with neutral stimuli. In addition, depressive episodes in BD patients showed lower left dorsal anterior midcingulate cortex (dAMCC) activation during visuospatial n-back task with neutral stimuli (Bertocci et al., 2011). In contrast, a different brain activation pattern was reported during n-back tasks with emotional auditory-verbal stimuli. Such as greater left dorsolateral prefrontal cortex (DLPFC) and dACC activation were found when negative valence stimuli were presented (Deckersbach et al., 2008). Therefore, it was suggested that updating was influenced by emotional factors.

According to processing efficiency theory (Eysenck and Calvo, 1992), high levels of anxiety reduce the efficiency of cognitive processing, specifically the central executive of the WM system (Derakshan et al., 2009), confirming the influence of emotions on updating. Besides there are three major control functions of the central executive: inhibition, shifting, and updating (Miyake et al., 2000), study by Eysenck et al. (2007), based on attentional control theory showed that anxiety impairs two major functions of the central executive: negative attentional control (inhibition function) and positive attentional control (shifting function).

In addition, to the decrease activation in dorsolateral prefrontal cortex, parietal alterations in manic, and depressive episodes of BD patients were reported during visuospatial tasks (Pomarol-Clotet et al., 2014). Even in euthymic patients the brain activation pattern varied depending on the mood valences (Malhi et al., 2007). Thus, there was more activity in dACC and amygdala regions over negative valences and a greater connection between dACC and amygdala regions in positive valences (Mullin et al., 2012). It is consistent with the Somatic Marker hypothesis that supports physiological influence on emotional responses through the central executive (Damasio et al., 1991; Damasio, 1998). It was also verified the existence of autonomic changes associated with emotional reactivity during cognitive tasks (Muhtadie and Johnson, 2015). Then, by inference it seems that Hedonic Detector is also affected in WM tasks requiring information updating.

Remarkably, few studies assessed WM performance in more than one mood episode (Fleck et al., 2005; Pomarol-Clotet et al., 2014). Only four papers carried out mood induction prior performing tasks (Malhi et al., 2007; Deckersbach et al., 2008; Bertocci et al., 2011; Mullin et al., 2012) and just the research of autonomic response used a verbal stressor during the test (Muhtadie and Johnson, 2015). In this way, it is difficult to generalize the results for the second objective of this review. In addition, many studies selected BD I patients samples (Fleck et al., 2005; Malhi et al., 2007; Deckersbach et al., 2008; Drapier et al., 2008; Bertocci et al., 2011; Levy et al., 2011; Mullin et al., 2012; Gruber et al., 2013), and few studies assessed BD II subjects. Most papers did not differentiate subtypes of the disorder.

Despite Baddeley's model is the most commonly used theory in WM researches (Malhi et al., 2007; Thompson et al., 2007; Barrett et al., 2008; Dittman et al., 2008; Roiser et al., 2009; Liu et al., 2010; Levy et al., 2011; Gruber et al., 2013; Lee et al., 2013; Miguélez-Pan et al., 2014; Russo et al., 2014; Bauer et al., 2015; Muhtadie and Johnson, 2015), the discrepancies between studies may be explained by the new appearance neuroscience technologies that demand designed tasks such as n-back tasks (Deckersbach et al., 2008; Drapier et al., 2008) to investigate state-based models (D'Esposito, 2007). It appears that authors rarely made the connection between theoretical models and cognitive tasks. Then, despite the fact of some studies confirmed emotion and WM association in depressive or manic BD patients (Malhi et al., 2007; Deckersbach et al., 2008; Mullin et al., 2012; Pomarol-Clotet et al., 2014; Muhtadie and Johnson, 2015), the Hedonic Detector component was not considered in these selected articles of this review.

Once n-back tasks and complex span tasks apparently evaluate different processes (Ribeiro et al., submitted; Kane et al., 2007), WM researches should consider the differences between tasks and how they affect the result explanations (Redick and Lindsey, 2013; Remoli and Santos, 2017). That seems to be crucial for explaining the outcomes and for the replication of future studies (Ribeiro et al., submitted). Further restriction of this review is that diagnoses of BD suffered some changes from DSM-IV (American Psychiatric Association, 2000) to DSM-V (American Psychiatric Association, 2013). This fact could lead some methodological differences in selected studies. However, it was not the purpose of this work to analyze diagnoses differences of BD.

### Relationship among WM and other clinical features

Only article by Liu et al. (2010) compared BD I and BD II subjects. They showed that neural activation patterns were different between subgroups of the disorder during auditory-verbal information processing tasks. BD type I patients reported lateralized changes in the right hemisphere and more cognitive deficits while BD type II subjects revealed more distributed lower performance associated with cognitive and emotional processes.

In addition to this other evidence suggest that BD I and II patients present different neuropathological substrates in terms of the loss of bundle coherence or the disruption of fiber tracts (Liu et al., 2010), BD type I and II also exhibit heterogeneous clinical presentations and cognitive functions. Besides BD I patients manifested more cognitive dysfunction in verbal learning, recall, recognition, and set-shifting compared to bipolar II patients (Simonsen et al., 2008), it has been suggested that both suicide and attempted suicide are more common in BD II disorder than in BD I disorder (Jamison, 2000; Rihmer and Kiss, 2002; Hawton et al., 2005).

Article by Levy et al. (2011) showed that BD I patients readmitted to the hospital after 3-month had more psychotic episodes, lower level of global functionality, severe mood episodes, and lower scores for executive function, attention, and WM, visual, and verbal episodic memory performance comparing to outpatients care. While WM low score in BD type I samples were suggested in some studies (Drapier et al., 2008; Levy et al., 2011; Pomarol-Clotet et al., 2014), WM deficits were not found in the majority of BD II sample (Roiser et al., 2009). BD I hospitalized in mania/mixed with psychotic symptoms group also reported lower scores for recognition effectiveness and directed-forgetting effectiveness than expected (Fleck et al., 2005).

Although, epidemiological studies with non-clinical populations have suggested psychotic experiences as a predictor of suicidal behavior (Nishida et al., 2010; DeVylder et al., 2015) and study by Finseth et al. (2012) showed that suicidal attempt are more common in patients with mood disorders with psychotic features, this has not been a consensus in all studies. Besides psychotic symptoms are more common in BD type I patients, a recent study by Gesi et al. (2016) showed that psychotic features, as evaluated upon the presence of delusions or hallucinations, are not associated with suicidality among subjects with BD I, suggesting that suicide behavior is more common in BD type II disorder.

It is important to note that anxiety (Derakshan et al., 2009) and even mood (Derakshan et al., 2009) could modulate cognitive performance as, for example, attentional control. Future studies should address the role of mood states and even positive and negative emotions in working memory in BD patients to explore the role of hedonic detector.

### Emotional states and working memory: hedonic detector and its implication to cognition in mood disorders

In fact, the connection between emotional states and working memory plays a role in the measurement instruments and in theoretical framework as well. For instance, the Somatic Marker hypothesis presented by Damasio et al. (1991) describes a mechanism for interaction between emotion and cognition in trial processes and decision-making (Bechara and Damasio, 2002, 2005; Bechara et al., 2005; Verdejo-García and Bechara, 2009). Damasio even states that the executive component of WM could be relevant in this process. Baddeley's model of WM explicitly addressed the interference of emotions in WM information processing, by its new component, the Hedonic Detector (Baddeley, 2007). The Hedonic Detector works as a neutral point that varies between positive and negative valences in response to environmental stimuli. It establishes a mean value between stimulus and information retained in the WM to enable choices of future actions (Baddeley, 2007). In this perspective, improper adjustment of the neutral point could enhance the appearance of pathological affective episodes, for instance, the Bipolar Disorder (BD).

Deficits in WM processing have been consistently reported in euthymic patients (MacQueen et al., 2005; Thompson et al., 2007; Daglas et al., 2015). Another study also observed that euthymic BD type I had worse performance on visuospatial tasks compared to healthy subjects (Farahmand et al., 2015). Although, there is no consensus in the literature, these findings suggest that lower performance in WM could result in the intensification of emotional valences. In other words, the presence of WM deficits, could be considered a primary trace of BD, beyond state variables (Gruber et al., 2009; Kurtz and Gerraty, 2009). However, this particular issue remains controversial due to the diversity of methodologies used across the studies. Apart from the prevalence of within-subjects design, studies rarely made the connection between theoretical models and cognitive tasks, hindering the comprehension about WM components.

In line with this argument, a review by Baddeley (2013) showed that in depressive patients negative mood influences hedonic judgment, explaining the trend of the negative perception of the situations in this clinical population. However, there is no significant evidence in respect to the influence of positive mood in hedonic detection system. Hypothetically, assuming that the neutral point in BD patients corresponds to euthymic phase, the exacerbated positive and negative valences would account for euphoric or depressed mood, respectively (Baddeley, 2007, 2013). As emotion in BD is deregulated, the study of this disorder seems to be necessary to understand the influence of improper adjustment of the Hedonic Detector neutral point in WM.

## Conclusion

In conclusion, BD mood episodes were associated with both WM processes (updating and serial recall), cognitive lower performance persist even in remission of symptoms. This evidence suggests that BD patients have deficits in monitoring content in WM. But the data at this point is so different across studies that it does not seem prudent to generalize conclusions. Considering that WM deficit apparently is stage-independent in BD patients (Gruber et al., 2009; Kurtz and Gerraty, 2009), future studies should evaluate the convergence of Damasio's Somatic Marker hypotheses and Baddeley's Hedonic Detector in BD. In other words, how the neutral point is deregulated by the interaction between environmental stimuli and mood episodes and affect WM processes.

## Author contributions

Conception or design of the work: CS and FS. Data collection: CS. Data analysis and interpretation: CS and FS. Drafting the article: CS, FS, PA, and LM. Critical revision of the article: FS, PA, and LM. Final approval of the version to be published: CS, FS, PA, and LM. Agreement to be accountable for all aspects of the work in ensuring that questions related to the accuracy or integrity of any part of the work are appropriately investigated and resolved: CS, FS, PA, and LM.

### Conflict of interest statement

The authors declare that the research was conducted in the absence of any commercial or financial relationships that could be construed as a potential conflict of interest.
